# Responses of Wheat Yield, Macro- and Micro-Nutrients, and Heavy Metals in Soil and Wheat following the Application of Manure Compost on the North China Plain

**DOI:** 10.1371/journal.pone.0146453

**Published:** 2016-01-15

**Authors:** Fan Wang, Zhaohui Wang, Changlin Kou, Zhenghua Ma, Dong Zhao

**Affiliations:** 1 State Key Laboratory of Crop Stress Biology in Arid Areas, Northwest Agricultural and Forestry University, Yangling, Shaanxi, China; 2 Key Laboratory of Plant Nutrition and the Agri-environment in Northwest China, Ministry of Agriculture, College of Natural Resources and Environment, Northwest Agricultural and Forestry University, Yangling, Shaanxi, China; 3 Institute of Plant Nutrition and Resource Environment, Henan Academy of Agricultural Sciences, Zhengzhou, China; 4 Henan Academy of Agricultural Sciences/Key Laboratory of Animal Immunology of the Ministry of Agriculture/Henan Provincial Key Laboratory of Animal Immunology, Zhengzhou, China; Chinese Academy of Sciences, CHINA

## Abstract

The recycling of livestock manure in cropping systems is considered to enhance soil fertility and crop productivity. However, there have been no systematic long-term studies of the effects of manure application on soil and crop macro- and micro-nutrients, heavy metals, and crop yields in China, despite their great importance for sustainable crop production and food safety. Thus, we conducted field experiments in a typical cereal crop production area of the North China Plain to investigate the effects of compost manure application rates on wheat yield, as well as on the macro-/micro-nutrients and heavy metals contents of soil and wheat. We found that compost application increased the soil total N and the available K, Fe, Zn, and Mn concentrations, whereas the available P in soil was not affected, and the available Cu decreased. In general, compost application had no significant effects on the grain yield, biomass, and harvest index of winter wheat. However, during 2012 and 2013, the N concentration decreased by 9% and 18% in straw, and by 16% and 12% in grain, respectively. With compost application, the straw P concentration only increased in 2012 but the grain P generally increased, while the straw K concentration tended to decrease and the grain K concentration increased in 2013. Compost application generally increased the Fe and Zn concentrations in straw and grain, whereas the Cu and Mn concentrations decreased significantly compared with the control. The heavy metal concentrations increased at some compost application rates, but they were still within the safe range. The balances of the macro-and micro-nutrients indicated that the removal of nutrients by wheat was compensated for by the addition of compost, whereas the level of N decreased without the application of compost. The daily intake levels of micronutrients via the consumption of wheat grain were still lower than the recommended levels when sheep manure compost was applied, except for that of Mn.

## Introduction

Livestock manure is an organic fertilizer that contains organic nutrients such as amino acids, nucleic acids, sugars, and vitamins, but it is also a valuable source of organic matter, nitrogen, phosphorus, potassium, and some micronutrients [1]. The recycling of livestock manure in cropping systems is considered to enhance soil fertility and crop productivity [2] by ameliorating soil properties [3], increasing the capacity for nutrient retention [4], and by gradually improving the soil macronutrient status [5], which also determines the availability of micronutrients to plants [6].

The North China Plain is one of the world’s most important agricultural regions, where 35 million ha of croplands produce about 61% of the wheat used in China [7], as well as supplying 25%, 45%, and 33% of the meat, eggs, and milk, respectively, used in the country [8]. Therefore, the production of livestock and poultry manure amounted to almost 4.8 billion tons in 2010, which was 25% of the total in China [9]. However, over the past three decades, due to the rapid development of the economy, extra labor demands, and high cost inefficiencies, the traditional cropping practice with organic manure application has almost disappeared, where chemical fertilizers are now used widely in crop production as the main sources of crop nutrients. For example, the application rate for manure was around 102 kg N ha^–1^ a^–1^ in the winter wheat-summer maize rotation system in Shandong province during 1999 [10], whereas it declined to 53 kg N ha^–1^ a^–1^ during 2006 [11]. Thus, the input of nutrients with manure into farmland had gradually decreased, thereby decelerating the accumulation of organic matter and the soil organic matter (SOM) contents are now lower than 10 g kg^–1^ [12]. Previous research into soil degradation on the North China Plain by Yang and Janssen [13] showed that the continuous application of excess fertilizer N led to an imbalance between SOM inputs and SOM outputs, thereby affecting the accumulation and consumption of SOM in the soil over time, which would finally result in nutrient deficiencies in cultivated land. Obviously, this nutrient imbalance is a growing threat to food security on the North China Plain, where most of the regions are dependent on agriculture.

In recent years, manure application and its importance for sustainable agricultural development have attracted increasing attention [14]. Research has shown that the combined use of organic manure and chemical fertilizers may be an effective approach for combating nutrient depletion and for promoting sustainable crop production [15]. Jiang et al. [16] stated that manure application can increase the crop yield and SOM, as well as improving the soil quality. Abbasi and Tahir [17] demonstrated that the application of farmyard manure/poultry manure and other organic substrates either alone or in combination with inorganic fertilizers had positive effects on wheat productivity. However, many studies have also shown that manure application changes the levels of soil macronutrients and their availability, which may affect the soil micronutrient levels [18]. Long-term organic fertilization using manure or manure plus NPK fertilizer can significantly increase the soil organic C, total N, NH_4_^+^–N, total S [19], and soil-available P levels [5]. Thus, the concentrations of available Zn, Fe, and Mn in the soil were found to increase significantly with the organic matter contents [18]. In particular, the presence of PO_4_^2−^, H_2_PO_4_^−^, or HPO_4_^2−^ anions in soil solution can lead to the precipitation of Zn_3_(PO_4_)_2_ with Zn ions, thereby immobilizing Zn and decreasing its mobility in the soil with increasing manure application [6,18]. Some studies have even shown that manure can increase the accumulation of heavy metals in soil. Thus, Ju et al. [20] showed that the mean Cd concentration in vegetable soils was 2.8 times that in soils used for wheat-maize rotations due to the excessive application of fertilizers and manure during greenhouse vegetable production in northeast China. Rezig et al. [5] showed that the recycling of organic materials, especially biosolids (e.g., livestock manures), should be conducted carefully to avoid the accumulation of toxic elements and environmental contamination. Dong et al. [21] stated that manure was the main source of Cu, Zn, Cd, Ni, Pb, and Cr in a wheat-maize field system in north China, where it accounted for 86.1%, 83.8%, 76.6%, 72.5%, 64.3%, and 46.3% of the total external input for these heavy metals, respectively.

However, there is a lack of systematic, comprehensive, and long-term monitoring of the effects of manure application on soil nutrients, heavy metals, crop yields, and soil quality on the North China, which are of great importance for sustainable crop production and food safety. Therefore, the objectives of the present study were: (1) to examine the effects of organic manure application on wheat yields; (2) to determine the changes in the levels of macronutrients and microelements in soil and wheat; and (3) to assess the accumulation of heavy metals in soils and wheat on a fluvo-aquic soil on the North China Plain.

## Materials and Methods

### Ethics Statement

This study was performed on private land and the owner of the land granted permission to conduct the experiments before they commenced. No specific permission was required for the experimental locations or activities because the experimental activities had no negative impacts on the farmer, other people, the land, and the environment. The field studies did not involve endangered or protected species, and we only used sheep manure compost, which also contained bedding straw. The sheep manure compost was prepared and transported both manually and using machinery from the sheepfold to the field, and this study did not involve the sacrifice of any sheep. No approval was required from the Institutional Animal Care and Use Committee because the study did not involve animal sacrifice and we only used sheep manure. All of the sampling and/or experimental procedures were reviewed and specifically approved as part of the field study permission obtained from the farmer.

### Site description

**Fig 1:**
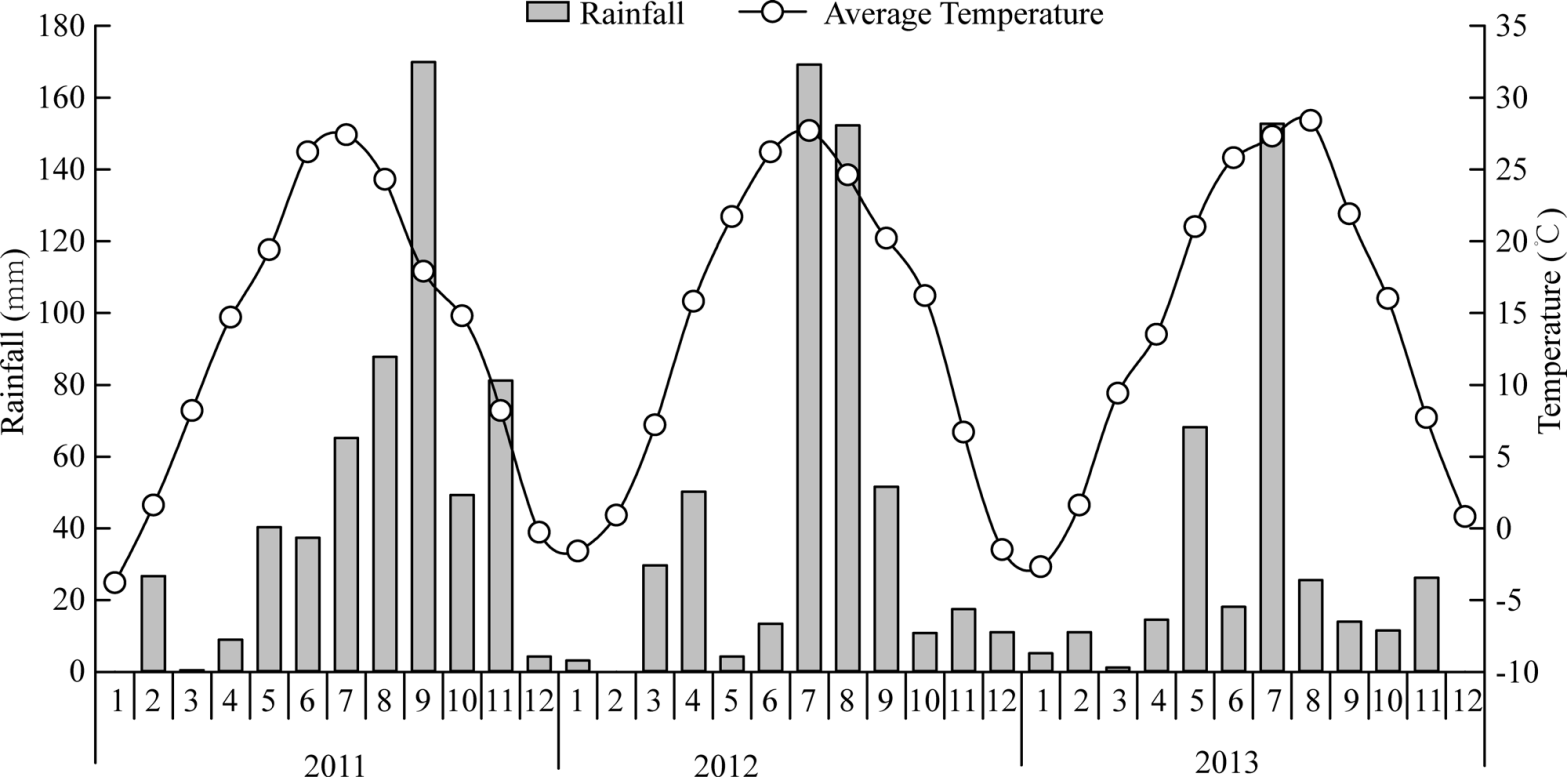
Rainfall and Average Temperature during the Experimental Years at Xun County on the North China Plain.

The field experiment was conducted during the wheat-growing seasons from 2011 to 2013 in Xun County, Henan Province, China (35°54′N, 114°11′E). This region has a warm temperate, sub-humid, continental monsoon climate. The mean annual rainfall and temperature are 478 mm and 13.7°C, respectively, and the annual precipitation occurs mainly between June and October. The monthly mean temperature and monthly precipitation during the experimental period are shown in Fig 1. The soil is a Eutric Cambisol with a clay loam texture and the chemical properties of the tested soil (0–30 cm) when the study was initiated in 2005 were: pH of 7.8, 8.9 g kg^–1^ organic C, 0.7 g kg^–1^ total N, 75.3 mg kg^–1^ available N (alkali-hydrolysable N), 6.5 mg kg^–1^ Olsen-P, 109.5 mg kg^–1^ available K; available Cu, Zn, Fe, and Mn were 1.9, 2.8, 16.3, and 6.2 mg kg^–1^, respectively; and the levels of the heavy metals Pb, Cd, and Cr were 3.5, 0.3, and 55.9 mg kg^–1^, respectively.

### Experimental design

The study was carried out on a private land, and the owner of the land had permitted it to be conducted on his land at the start of the experiment. The experiment was conducted using a randomized complete block design with three replicates, where the plot size measured 16.8 m^2^ (4.2 × 4 m). Five treatments used sheep manure compost (Table 1) at application rates designated as 0 (no-manure control, M0), 7500 (M1), 15000 (M2), 22500 (M3), and 30000 kg ha^–1^ (M4) on the basis of 225 kg ha^−1^ N fertilizer (urea, 46%), 120 kg ha^–1^ P_2_O_5_ fertilizer (superphosphate, 16%), and 120 kg ha^–1^ K_2_O fertilizer (potassium chloride, 60%) in the wheat season. Before sowing the winter wheat (*Triticum aestivum L*.), manure and P and K fertilizer were applied as basal fertilizers, while 60% of the N fertilizer was applied as a basal dressing and 40% as a top-dressing at the reviving stage. Winter wheat (*Triticum aestivum L*.) was sown on October 12, 2011 and October 13, 2012 after the summer maize (*Zea mays L*.*)* harvest, with a row spacing of 18 cm and a seeding rate of 180 kg ha^−1^, before harvesting in the following year on June 7, 2012 and June 9, 2013. The wheat cultivar used was Zhoumai 22. Before sowing the winter wheat, summer maize was harvested and the straw was returned and incorporated into the soil using a rotary tiller. Winter wheat was irrigated with 90 mm water at the elongating and flowering stages. The other field management practices were the same as those employed by local farmers.

**Table 1:** Nutrient and Heavy Metal Concentrations in the Sheep Manure Compost Used in the Experiment.

Organic matter	Total nutrients (g kg^−1^)			Available nutrients (mg kg^−1^)				Total metals (mg kg^−1^)		
(g kg^−1^)	N	P	K	Cu	Zn	Fe	Mn	Pb	Cd	Cr
354	20	5.2	15.5	4.0	68.9	1248.9	45.6	15.8	0.7	8.9

### Wheat sampling and analysis

When the wheat plants had reached maturity, two 1-m long rows were harvested manually from each plot, and the roots were cut off at the connection between the root and stem. The aboveground parts of each sample were divided into ears and straw (including the stem and its leaves). After air-drying, the ears were threshed and the grain, glumes, and straw were weighed. The harvest index (HI) was calculated as the fraction of grain dry matter divided by the total aboveground biomass on a per hectare basis. Subsamples comprising 100 g grain and 50 g glumes or straw were oven-dried to constant weight at 60°C. The dried samples were crushed with a stainless steel grinder and stored in sealed plastic bags for chemical analysis.

The crushed wheat grain and straw samples were digested with concentrated sulfuric acid (H_2_SO_4_, AR, 98%) and hydrogen peroxide (H_2_O_2_, GR, ≥30%) [22]. The N concentration was determined by the Kjeldahl method. The P concentration was measured using an AutoAnalyzer 3 (AA3, SEAL Company, Germany) continuous flow analyzer and the total K was analyzed by a flame photometer. Elemental analysis of Fe, Mn, Cu, Zn, Pb, Cd, and Cr in wheat grain and straw was performed by inductively coupled plasma-atomic emission spectroscopy.

### Soil sampling and analysis

Soils were sampled from depths of 0–30 cm at wheat sowing and the harvest during each year. Three soil cores were taken from each plot using an auger (inner diameter = 4.0 cm), where the soil samples from the same plot were mixed and sealed immediately in a marked plastic bag. Soil samples were air-dried before analyzing the soil pH, soil organic C, total N, available P and K, as well as the available Cu, Zn, Fe, and Mn, and heavy metals, i.e., Pb, Cd, and Cr.

Soil pH was determined using a pH electrode at a soil to water ratio of 1:2.5. Soil organic C was measured using the potassium dichromate method. Total N was measured using the Kjeldahl method. Available P was extracted with 0.5 M NaHCO_3_. Available K was extracted with 1.0 M NH_4_OA [22]. Micronutrients and heavy metals were determined by the diethylene triamine pentaacetic acid extraction-inductively couple plasma method (Prodigy7, Leeman, USA).

### Statistical analysis

Data were analyzed using one-way analysis of variance with SPSS 18.0 (SPSS Inc., Chicago, USA). Means among treatments in the same year were compared by the least significant difference (LSD) test at *P* < 0.05.

## Results

### Grain yield, biomass, and HI of wheat

The application of sheep manure compost had no significant effects on biomass in either of the two years, or on the wheat grain yield in most cases (Table 2). The average wheat grain yield and biomass with manure application were 7939 and 17791 kg ha^−1^ in 2012, and 8548 and 20186 kg ha^−1^ in 2013. The grain yield and the biomass were higher in 2013 than in 2012.

**Table 2:** Effects of Different Compost Manure Application Levels on the Wheat Grain Yield, Aboveground Biomass, and Harvest Index after Harvesting Winter Wheat in the Two Experimental Years of 2011–2012 and 2012–2013.

Treatment	Manure	Grain yield(kg ha^–1^)		Biomass (kg ha^–1^)		Harvest index(%)	
	rate(kg ha^–1^)	2012	2013	2012	2013	2012	2013
M0	0	8356a	8727a	19825a	21492a	42b	41a
M1	7500	7763a	8060b	16768a	19602a	46a	42a
M2	15000	8412a	8986a	19343a	21121a	43ab	43a
M3	22500	7781a	8393ab	17361a	19269a	45ab	44a
M4	30000	7800a	8755ab	17694a	20749a	44ab	42a

For each parameter, means followed by different letters are significantly different according to LSD (*P* < 0.05).

HI did not differ significantly among treatments in 2013, but it was significantly higher with M1 (46%) than M0 (42%) in 2012 (Table 2).The average HI with the manure applications was 45% in 2012, which was higher than that in 2013 (43%).

### Macronutrients in soil and wheat

#### Macronutrients in soil

The soil total N concentration ranged from 0.8 to 1.1 g kg^–1^ in 2012 and from 0.8 to 1.0 g kg^–1^ in 2013 across different treatments (Table 3). Compared with the control, total N was increased significantly by compost application at the higher level, where it increased by 17% with M3 in 2012 and by 11% with M4 in 2013.

**Table 3:** Effects of Different Compost Manure Application Levels on the Soil Total N, Available P, and Available K after Harvesting Winter Wheat during the Two Experimental Years of 2011–2012 and 2012–2013.

Treatment	Manure	Total N(g kg^–1^)		Available P (mg kg^–1^)		Available K(mg kg^–1^)	
	Rate (kg ha^–1^)	2012	2013	2012	2013	2012	2013
M0	0	0.9b	0.9bc	14.8ab	14.4a	155.6b	164.7b
M1	7500	0.8b	0.8c	7.7c	7.8b	144.8b	194.1ab
M2	15000	0.9b	0.8c	10.5bc	12.0a	149.8b	210.2a
M3	22500	1.1a	1.0ab	16.2a	14.7a	218.8a	212.2a
M4	30000	1.0a	1.0a	14.4ab	12.8a	228.0a	231.1a

For each parameter, means followed by different letters are significantly different according to LSD (*P* < 0.05).

The variation in the available P in soil exhibited different trends compared with the total N. Under the different fertilization treatments, the changes in soil available P were different, where there was a decrease with M1, but no change with the M2, M3, and M4 treatments.

Different compost application rates had significant effects on the soil available K (Table 3). Compared with M0, the soil available K increased by 41% with M3 and by 47% with M4 in 2012, and by 18%, 28%, 29%, and 40% with M1, M2, M3, and M4, respectively, in 2013. The average available K levels with all of the compost treatments were 185 and 212 mg kg^−1^ in 2012 and 2013, respectively, which were 19% and 29% higher compared with those under M0.

#### Macronutrients in wheat straw and grain

The nitrogen concentration in wheat straw decreased in most cases when compost was applied, except for M2 in 2012 and M4 in 2013. The nitrogen concentrations were 6.4 and 6.9 g kg^–1^ with M0 during 2012 and 2013, respectively, but in the same two years, the average concentrations under the compost applications were 5.9 and 5.7 g kg^–1^ (Table 4), i.e., reductions of 9% and 18% compared with M0. Similar results were obtained when we tested the grain. The grain N concentrations with M0 in 2012 and 2013 were 27.1 and 24.7 g kg^–1^, respectively, whereas the average concentrations with the four compost applications were 22.7 and 21.8 g kg^–1^ in the same two years, i.e., reductions of 16% and 12%.

**Table 4:** Concentrations of N, P, and K in Wheat Straw and Grain after the Harvest of Winter Wheat under Different Compost Manure Application Rates during the Two Experimental Years of 2011–2012 and 2012–2013.

Treatment	Manure	Wheat straw						Wheat grain					
	rate(kg ha^–1^)	Total N (g kg^–1^)		Total P(g kg^–1^)		TotalK(g kg^–1^)		Total N(g kg^–1^)		Total P (g kg^–1^)		Total K(g kg^–1^)	
		2012	2013	2012	2013	2012	2013	2012	2013	2012	2013	2012	2013
M0	0	6.4b	6.9b	1.4c	1.3a	23.0a	38.8a	27.1a	24.7a	3.6c	4.1b	3.4ab	3.1c
M1	7500	5.5c	5.1d	2.1a	1.3a	18.9e	32.5b	25.0b	21.5c	5.0a	4.7a	3.4ab	3.5b
M2	15000	7.8a	6.2c	1.9a	1.2a	21.2b	30.7c	23.9c	21.2d	4.0b	3.6d	3.5a	3.6b
M3	22500	4.8d	4.1e	1.9ab	1.2a	20.3c	23.4e	21.7d	21.3d	4.1b	4.7a	3.4ab	3.6b
M4	30000	5.3c	7.2a	1.7b	1.2a	19.6d	28.4d	20.1e	23.0b	3.7c	3.8c	3.2b	3.8a

For each parameter, means followed by different letters are significantly different according to LSD (*P* < 0.05).

The straw P concentration increased significantly under the compost applications in 2012, but it did not increase continuously with the compost inclusion rate. The application of compost had no significant effect on the straw P in 2013. The straw P concentration was 2.1 g kg^–1^ with M1 in 2012, which was 47% and 22% higher than the concentrations with M0 and M4, respectively. Different results were obtained for the wheat grain P. The grain P concentration was 5.0 g kg^–1^ with M1 in 2012, which was 40%, 23%, 22%, and 35% higher than that with M0, M2, M3, and M4, respectively, and 4.7 g kg^–1^ in 2013 (Table 4), which was 13%, 31%, and 22% higher than that with M0, M2 and M4.

The K concentration in wheat straw was decreased by compost application over the two-year period, with average levels of 20 and 29 g kg^–1^ in 2012 and 2013 under the compost treatments, respectively, i.e., reductions of 13% and 26%. The application of manure compost significantly increased the K concentration in wheat grain during 2013 (Table 4), where the average with the compost treatments was 3.6 g kg^−1^, which was 17% higher than with the control. However, there was no significant difference among the treatments during 2012 compared to the control (Table 4).

### Micronutrients in soil and wheat

#### Micronutrients in soil

Compared with the M0 treatment, the application of compost decreased the concentration of soil available Cu, whereas the concentrations of Fe, Zn, and Mn increased (Table 5). At higher compost application rates, the available Fe increased in the soil, i.e., 15.6 and 13.7 mg kg^−1^ with M3 and M4 during 2012, respectively, which were 28% and 13% higher than that with M0, and 14.0 and 12.5 mg kg^−1^ during 2013, which were 23% and 10% higher than that with M0.

**Table 5:** Extractable Micronutrient Concentrations in the Soil after the Harvest of Winter Wheat under Different Compost Manure Application Rates during the Two Experimental Years of 2011–2012 and 2012–2013.

Treatment	Manure rate(kg	Available Fe (mg kg^–1^)		Available Zn (mg kg^–1^)		Available Cu (mg kg^–1^)		Available Mn (mg kg^–1^)	
	ha^–1^)	2012	2013	2012	2013	2012	2013	2012	2013
M0	0	12.2bc	11.4c	2.1ab	1.2c	1.3a	1.3a	6.9a	7.0b
M1	7500	10.8c	11.8bc	1.5b	1.5b	1.2ab	1.2b	7.0a	7.2b
M2	15000	12.4bc	12.3bc	2.0ab	1.7b	1.1ab	1.2b	7.2a	7.5b
M3	22500	15.6a	14.0a	2.2ab	2.2a	1.1b	1.2b	7.0a	8.2a
M4	30000	13.7ab	12.5b	2.2a	2.2a	1.1b	1.2b	7.0a	8.4a

For each parameter, means followed by different letters are significantly different according to LSD (*P* < 0.05).

The application of compost had significant effects on the available Zn concentration in both years (Table 5), where the maximum level was 2.2 mg kg^–1^ with M3 and M4 in 2013, which was 83% higher than that with the control. However, the application of compost decreased the available Cu compared with the control. The average available Cu concentrations in the soil under compost application during 2012 and 2013 were 1.1 and 1.2 mg kg^−1^, which were 14% and 11% lower than that with the control, respectively. The amounts of available Cu in the soil under different compost treatments did not differ significantly in either year (Table 5), whereas the amounts with the compost treatments and the control differed.

The soil available Mn concentration was increased by the application of compost only in the second year, where the average under the compost treatments was 7.8 mg kg^–1^, which was 11% higher than that with the control.

#### Micronutrients in wheat straw and grain

In general, the application of manure compost increased the concentrations of Fe and Zn in wheat straw and grain, but the Cu and Mn concentrations tended to decrease with the compost treatments compared with the corresponding control in each year, except the Cu level in straw increased during 2013 (Table 6).

**Table 6:** Concentrations of Fe, Zn, Cu, and Mn in Wheat Straw and Grain after the Harvest of Winter Wheat under Different Compost Manure Application Rates during the Two Experimental Years of 2011–2012 and 2012–2013.

**Treatments**	Wheat straw								Wheat grain							
	Fe(mg kg^-1^)		Zn(mg kg^-1^)		Cu(mg kg^-1^)		Mn(mg kg^-1^)		Fe(mg kg^-1^)		Zn(mg kg^-1^)		Cu(mg kg^-1^)		Mn(mg kg^-1^)	
	2012	2013	2012	2013	2012	2013	2012	2013	2012	2013	2012	2013	2012	2013	2012	2013
M0	209.7c	264.7b	8.9a	8.4bc	6.3a	4.5b	28.5a	34.0b	25.6b	19.6b	12.8c	13.4b	3.3ab	3.6a	31.7a	32.8a
M1	237.5c	352.9ab	10.0a	6.6c	5.9ab	8.0a	27.9ab	32.0ab	24.3b	24.3ab	12.2d	14.9ab	2.7b	2.9ab	28.0b	22.3c
M2	305.2b	361.6a	7.1a	12.2a	4.0c	6.4ab	27.4ab	31.2ab	42.1a	25.5ab	13.9b	15.1ab	3.7a	2.9ab	29.9ab	22.0c
M3	342.2ab	381.8a	7.4a	11.0ab	5.0abc	8.0a	26.2ab	30.6ab	45.6a	33.5a	14.9a	15.7ab	2.8b	2.7b	29.0ab	23.9bc
M4	378.1a	406.0a	7.6a	11.7a	4.4bc	5.0b	25.7b	27.1b	47.0a	34.1a	15.0a	16.3a	3.0ab	2.9b	30.0ab	24.9b

For each parameter, means followed by different letters are significantly different according to LSD (*P* < 0.05).

For wheat straw, the average Fe concentrations under all of the compost treatments were 315.8 and 375.6 mg kg^–1^ during 2012 and 2013, respectively, while the average levels were 8.6 and 11.2 mg kg^–1^ for Zn, 4.8 and 6.9 mg kg^–1^ for Cu, and 26.8 and 30.2 mg kg^–1^ for Mn. In wheat grain, the average concentrations during 2012 and 2013 were 39.8 and 29.4 mg kg^–1^ for Fe, 14.0 and 15.5 mg kg^–1^ for Zn, 3.1 and 2.9 mg kg^–1^ for Cu, and 29.2 and 23.3 mg kg^–1^ for Mn, respectively. Compared with the corresponding controls, the compost treatment increased the Fe concentrations in wheat straw and grain by 46% and 53%, respectively, over the two years, as well as enhancing the Zn concentration by 12% in the second year. By contrast, the application of compost decreased the Mn concentration in wheat straw and grain by 9% and 18%, respectively, and reduced the grain Cu concentration by 14% over the two-year period.

### Pb, Cd, and Cr concentrations in soil and wheat

#### Pb, Cd, and Cr concentrations in soil

In general, the concentration of Cd in the soil was not affected by the application of compost, where the average levels were 0.13 and 0.17 mg kg^–1^ during 2012 and 2013, respectively, although there was a significant increase to 0.18 mg kg^–1^ with M3 during 2013 (21%) compared with the control.

The compost treatment had significant effects on the soil Cr during 2012 (Table 7). The average soil Cr concentration with the compost treatments was 55 mg kg^–1^ in 2012, which was 7% higher than the control. During 2013, the soil Cr concentration was not affected by the application of compost.

**Table 7:** Concentrations of Heavy Metals at Soil Depths of 0–30 cm under Different Compost Manure Application Rates during the Two Experimental Years of 2011–2012 and 2012–2013.

Treatment	Manure	Cd (mg kg^–1^)		Cr (mg kg^–1^)		Pb (mg kg^–1^)	
	rate(kg ha^–1^)	2012	2013	2012	2013	2012	2013
M0	0	0.14ab	0.15b	52.1c	54.3ab	4.4c	6.5ab
M1	7500	0.09b	0.17ab	56.8a	56.5a	3.2d	4.4b
M2	15000	0.13ab	0.17ab	56.3ab	51.6b	5.6b	6.0ab
M3	22500	0.15ab	0.18a	55.1ab	54.3ab	3.5d	5.2ab
M4	30000	0.16a	0.17ab	54.6b	52.5b	7.1a	6.9a

For each parameter, means followed by different letters are significantly different according to LSD (*P* < 0.05).

Compared with the control, the soil Pb was increased significantly with the compost applications, especially at higher rates, i.e., 7.1 mg kg^−1^ with M4 during 2012, which was 61% higher than that with M0, and 6.9 mg kg^−1^ during 2013, which was 7% higher.

#### Pb, Cd, and Cr concentrations in wheat straw and grain

The concentrations of Cd and Pb in wheat straw or grain were not detected (data not shown), which indicated that they were not affected by the application of sheep manure compost. However, the concentrations of Cr in wheat straw and grain were increased significantly by the application of manure compost (Table 8).

**Table 8:** Concentrations of Cr in Wheat Straw and Grain after the Harvest of Winter Wheat under Different Compost Manure Application Rates during the Two Experimental Years of 2011–2012 and 2012–2013.

Treatment	Manure	Wheat straw Cr (mg kg^–1^)		Wheat grain Cr (mg kg^–1^)	
	rate(kg ha^–1^)	2012	2013	2012	2013
M0	0	0.13c	0.17b	0.07c	0.08b
M1	7500	1.00b	1.00a	0.30b	0.42a
M2	15000	1.01b	1.00a	0.49ab	0.66a
M3	22500	1.08ab	1.10a	0.53ab	0.56a
M4	30000	1.17a	1.12a	0.68a	0.61a

For each parameter, means followed by different letters are significantly different according to LSD (*P* < 0.05).

Under the compost treatments, the average Cr concentrations in wheat straw were 1.07 and 1.05 mg kg^−1^ during 2012 and 2013, respectively. The average Cr concentrations in wheat grain were 0.50 and 0.56 mg kg^−1^ during 2012 and 2013, respectively. Thus, compared with the corresponding controls, the compost treatments increased the average Cr concentrations in wheat straw and grain by 6.3 and 5.9 times, respectively, over the two years.

## Discussion

### Grain yield of winter wheat

The application of sheep manure compost had no significant effects on the wheat grain yield during our two-year experiments, except with M1 in 2013, where the grain yield was reduced by 8%. The HI was increased only with M1 during 2012, but it was not affected by the application of compost. Thus, the nutrients from the soil and chemical fertilizers were already sufficient to meet the nutritional needs of the plants cultivated, as described previously [6]. Similar results were also reported by Abbasi and Tahir [17]. The wheat grain yield and biomass under different compost treatments were higher during the second year compared with the first year (7% and 12%, respectively), which might have been due to the 48% increase in rainfall during April and June (Fig 1) in the second year.

### Macronutrients in soil and winter wheat

Our results showed that the application of compost did not affect the soil total N at low rates, whereas it significantly enhanced the total N at high rates. Compared with the control, the total N concentration increased significantly by 17% with M3 in 2012 and by 11% with M4 in 2013. These results indicate that the addition of more organic C to the soil as manure compost increased the accumulation of organic N [23] and reduced its mineralization [24]. These results also agree with previous long-term experiments, which showed that organic fertilizers could have a priming effect on the soil total N [5,25]. In the present study, the soil available P was not affected significantly by most of the compost treatments, although it was decreased with M1. Similar results were obtained by Rezig et al. [5], where a possible explanation for this variability was that alkaline conditions induced P precipitation. In the present study, the application of compost markedly increased the availability of K in soils; however, this did not translate into a higher grain yield, thereby indicating that the soil K was not yield-limiting. The average available K levels under the compost treatments during 2012 and 2013 were 185 and 212 mg kg^−1^, respectively, which were 19% and 29% higher than the controls. These results are consistent with those obtained in a rainfed soybean-wheat system in India, where the combined application of NPK and manure increased the available K by 73% compared with the application of NPK alone [26]. Similar results were also obtained by Singh et al. [27]. Thus, organic manure has beneficial effects on the available K, where in addition to acting as a source of K, it also releases organic colloids with many cation exchange sites, thereby attracting K from the non-exchangeable pool and the applied K fertilizer, which ultimately improves its availability [28]. Our results showed that the application of compost increased the soil total N and the available K concentrations, whereas the available P was not affected in a fluvo-aquic soil, and the results obtained in a similar study based on a non-saline soil [5] were consistent with those of the present study.

In our experiments, the application of compost significantly influenced the concentrations of macronutrients in wheat straw and grain during both years, except for the straw P during 2013. Our results indicated that the average N concentrations in wheat straw and grain tended to decrease with the compost treatments, i.e., 9% and 18% reductions compared with the control in straw during 2012 and 2013, respectively, with 16% and 12% decreases in grain. The straw P concentration increased significantly in 2012, but it was not affected in 2013, whereas the grain P concentration generally increased with the compost treatments in both years. The K concentration in wheat straw decreased in both years, whereas the grain K concentration increased in 2013, but it was not affected in 2012. A higher compost C/N ratio and thus the immobilization of soil N may decrease the nitrogen content of wheat grain [5,17]. In the present study, the lowest amount of soil-available P occurred with the M1 treatment in both years, and the highest amounts of P uptake by wheat occurred with the same treatment. This indicates that the application of a low amount of organic fertilizer promoted P uptake by the crop. Similar results were obtained using cucumbers by Cui et al. [29]. The application of manure at low rates could lead to higher P uptake by wheat, which might enhance the release of P from sparingly soluble P forms (Ca_8_–P) for crop uptake, particularly in calcareous soils [30]. The decreased response of straw K to different manure treatments may be explained by the immediate requirement for nutrients in the early crop growth stages when induced by chemical fertilizers [31]. However, our results indicate that the application of manure significantly increased the grain K concentrations in the second year. Previous studies have also shown that combining chemical fertilizer with compost can significantly increase the concentration of K in wheat [32]. Similarly, Ning et al. [33] reported that the application of organic fertilizer contributed to the uptake of K in tobacco.

### Micronutrients in the soil and winter wheat

Long-term organic fertilization influences the concentrations of available micronutrients in the soil and plants. In our study, the application of compost decreased the concentration of available Cu in a fluvo-aquic soil, whereas the Fe, Zn, and Mn concentrations were increased compared with the M0 treatment, except for the available Fe and Zn in the soil with M1 during 2012. Similar results were reported by Li et al. [18], where it was suggested that the application of compost increased the organic matter content and it is known that Cu has a high affinity for organic matter [34]. By contrast, the results of a long-term field experiment showed that as the SOM contents increased, there were decreases in the mobility of Cu, Fe, and Mn in the soil solution, whereas that of Zn increased [6]. The mechanisms responsible for these effects were not investigated [19], but the variability may be due to the strong buffering capacity of the soil, or the micronutrient levels may depend on soil characteristics, including the pH, texture, organic matter, redox conditions [35], and the type and quantity of oxyhydroxides present, as well as the crop species considered [36].

The application of compost significantly affected the concentrations of micronutrients in wheat straw and grain. In general, the application of manure compost enhanced the Fe and Zn concentrations in wheat straw and grain, whereas the Cu and Mn concentrations tended to decrease under the compost treatments compared with the corresponding controls in each year, except for the straw Cu in 2013. However, the results obtained were different in a similar study conducted in Poland, where farmyard manure treatment decreased the Fe and Zn concentrations in wheat [6]. This difference indicates that the soil properties have significant effects on the absorption of Fe and Zn by plants. According to Campbell [37], the critical concentration of Fe in wheat plants during the vegetative phase is 25 mg kg^–1^, and the sufficiency concentration range for Fe in wheat flag leaves during the grain-filling stage is 30–200 mg kg^–1^. In the present study, the Fe concentrations in the wheat straw during the maturity stage ranged from 210 to 406 mg kg^–1^ (Table 6), which were similar to those found by Li et al. [18] and Zhang et al. [38]. Thus, our results and those of other studies indicate that organic fertilizer enhanced the concentration of Zn in wheat but decreased the concentration of Mn compared with the use of a chemical fertilizer [18]. This finding might be explained by the addition of organic material, which increased the SOM content, thereby affecting the transfer of Zn and Mn from the soil to plants. In the present study, manure compost increased the available Zn in the soil, which might be the main reason why the Zn concentration increased in the wheat straw and grain. However, the increased available Mn in the soil in the present study did not lead to increases in the concentration of Mn in the straw and grain, which requires further explanation. In addition, the decreased Cu content of wheat might have been caused by its reduced mobility in the soil. Similar results were also obtained by Singh et al. [27]. By contrast, the results that we obtained in 2013 showed that the straw Cu content increased. This inconsistency indicates that many factors may affect the uptake of Cu by plants, such as soil and climate conditions [39,40].

### Heavy metals in the soil and winter wheat

Surveys of the heavy metals in agricultural soils have been reported previously for different areas of China [41,42], which have shown that 10% of the agricultural soils in China are affected by heavy metal pollution [43]. In our study, the mean concentrations of the heavy metals ranged from 0.09 to 0.18 mg kg^–1^ for Cd, 51.6 to 56.8 mg kg^–1^ for Cr, and 3.2 to 7.1 mg kg^–1^ for Pb (Table 7). Compared with the different standards for heavy metal concentrations in soil (Table 9), the metal levels found in the soil in the study area were all lower than the world limits [44]. We also compared the mean concentrations of metals with the thresholds for elements in the natural background soil in China and the maximum allowable concentration for elements in agricultural soil in China (pH > 7.5, grade II), where all of the results obtained for heavy metals were much lower than the corresponding threshold values [45]. This indicates that the concentrations of the heavy metals were within the safe levels, although they were increased by the application of compost in some cases, so the soils in the present study can be considered “clean.”

**Table 9:** Standards for Heavy Metal Concentrations in Soil.

Metal	World limit^[42]^ (mg kg ^–1^)	Threshold in natural background soil in China^[43]^ (mg kg ^–1^)	Maximum allowable concentration in agricultural soil (pH > 7.5) in China^[43]^ (mg kg ^–1^)
Cd	0.35	0.2	1.0
Cr	70	90	250
Pb	35	35	350

Agricultural crops can readily take up and accumulate toxic metal elements from contaminated soils, which may pose a risk to human health through the food chain. In our study, under different treatments, the concentrations of Cd and Pb in wheat straw and grain were too low to be determined. These results indicate that the application of sheep manure did not cause Cd or Pb contamination of wheat. Our results also showed that the average Cr concentrations in wheat straw and grain across compost treatments over the two years were 1.06 and 0.53 mg kg^−1^, respectively. Compared to the recommended maximum tolerable levels (1.0 mg Cr kg^−1^) [46], the accumulation of Cr in wheat grain was lower than the threshold value. This suggests that the concentrations of Cr in the edible parts of wheat were within the safe level, but the rapid increases in the Cr contents of winter wheat straw and grain when sheep manure compost was applied requires further investigation.

### Nutrient balance and safety in the soil-plant system

#### Nutrient balance in the soil-plant system

According to the calculated macro-and micro-nutrients inputs due to the application of the sheep manure compost and their removal by wheat during 2011–2013, the macronutrient and micronutrient inputs with the compost-added treatments were sufficient to maintain the crop removal rates, except for N in the M1 treatment (Table 10). By contrast, the application of chemical NPK fertilizer alone resulted in obvious losses of these nutrients and several previous studies obtained similar results. Li et al. [47] reported a positive balance of the macronutrients in the soil under manure treatment in the central northern region of China. Zhang et al. [38] reported that the application of NPK alone resulted in micronutrient deficits, but not when the application of NPK was supplemented with manure. In the present study, the balance calculations for heavy metals showed that the application of compost increased the soil levels of Pb, Cd, and Cr (Table 10). However, the concentrations of Cd and Pb in wheat were still very low, or they could not be detected, and the accumulation of Cr in wheat grain was lower than the threshold value (Table 11), which indicates that the heavy metal levels in the compost were not a potential threat to the soil or the crop products.

**Table 10:** Balances of Macro-and Micro-nutrients, as well as Heavy Metals due to the Input from Sheep Manure Compost Application and Removal by Wheat Harvest in the Soil-plant System.

Element	Treatment	Input from manure compost(kg ha^−1^)	Removal by wheat harvest(kg ha^−1^)	Balance(input–output)
N	M0	0	442.0	–442.0
	M1	300	367.4	–67.4
	M2	600	391.6	208.5
	M3	900	347.6	552.4
	M4	1200	358.1	841.9
P	M0	0	65.9	–65.9
	M1	78	76.7	1.3
	M2	156	66.0	90.0
	M3	234	71.3	162.7
	M4	312	62.1	249.9
K	M0	0	55.5	–55.5
	M1	233	54.6	177.9
	M2	465	61.8	403.2
	M3	698	56.7	640.8
	M4	930	58.2	871.8
Fe	M0	0.00	0.38	–0.4
	M1	18.73	0.38	18.3
	M2	37.47	0.58	36.9
	M3	56.20	0.64	55.6
	M4	74.93	0.67	74.3
Zn	M0	0.00	0.22	–0.2
	M1	1.03	0.21	0.8
	M2	2.07	0.25	1.8
	M3	3.10	0.25	2.9
	M4	4.13	0.26	3.9
Cu	M0	0.00	0.06	–0.1
	M1	0.06	0.04	0.0
	M2	0.12	0.06	0.1
	M3	0.18	0.04	0.1
	M4	0.24	0.05	0.2
Mn	M0	0.00	0.55	–0.6
	M1	0.68	0.40	0.3
	M2	1.37	0.45	0.9
	M3	2.05	0.43	1.6
	M4	2.74	0.45	2.3
Cd	M0	0.00	0.00	0.0
	M1	0.01	0.00	0.0
	M2	0.02	0.00	0.0
	M3	0.03	0.00	0.0
	M4	0.04	0.00	0.0
Cr	M0	0.00	0.00	0.0
	M1	0.13	0.01	0.1
	M2	0.27	0.01	0.3
	M3	0.40	0.01	0.4
	M4	0.53	0.01	0.5
Pb	M0	0.00	0.00	0.0
	M1	0.24	0.00	0.2
	M2	0.47	0.00	0.5
	M3	0.71	0.00	0.7
	M4	0.95	0.00	0.9

Input from manure compost (kg ha^−1^) = added manure rate (kg ha^−1^) × element concentration (g kg^–1^)/1000

Removal by wheat harvest (kg ha^−1^) = grain yield (kg ha^−1^) × element concentration (g kg^–1^)/1000

Balance (kg ha^−1^) = input–output

**Table 11:** Estimated Human Health Risk due to the Intake of Micronutrients and Heavy Metals from the Consumption of Wheat Grain when Sheep Manure Compost was Applied.

Element	Treatment	Average concentration(mg kg^–1^)	Dietary intake(mg day^−1^)	Recommended daily intake, (male) ^[45]^ (mg day^−1^)	Recommended daily intake, (female)^[45]^ (mg day^−1^)	Recommended maximum tolerable levels ^[44]^ for heavy metal(mg kg^–1^)
Fe	M0	22.6	4.5	8	18	
	M1	24.3	4.9	8	18	
	M2	33.8	6.8	8	18	
	M3	39.6	7.9	8	18	
	M4	40.6	8.1	8	18	
Zn	M0	13.1	2.6	11	8	
	M1	13.6	2.7	11	8	
	M2	14.5	2.9	11	8	
	M3	15.3	3.1	11	8	
	M4	15.7	3.1	11	8	
Cu	M0	3.5	0.7	0.9	0.9	
	M1	2.8	0.6	0.9	0.9	
	M2	3.3	0.7	0.9	0.9	
	M3	2.8	0.6	0.9	0.9	
	M4	3.0	0.6	0.9	0.9	
Mn	M0	32.3	6.5	2.3	1.8	
	M1	25.2	5.0	2.3	1.8	
	M2	26.0	5.2	2.3	1.8	
	M3	26.5	5.3	2.3	1.8	
	M4	27.5	5.5	2.3	1.8	
Cd	M0	ND^a^				0.1
	M1	ND^a^				0.1
	M2	ND^a^				0.1
	M3	ND^a^				0.1
	M4	ND^a^				0.1
Cr	M0	0.1				1
	M1	0.4				1
	M2	0.6				1
	M3	0.5				1
	M4	0.6				1
Pb	M0	ND^a^				0.2
	M1	ND^a^				0.2
	M2	ND^a^				0.2
	M3	ND^a^				0.2
	M4	ND^a^				0.2

^a^ Not detected. The average concentrations of the micronutrients or heavy metals were averaged over the two experimental years under the same treatment.

The daily intake (DI*i*) of elements was calculated by: DI*i* = C*i*×Q, where C*i* is the concentration of element *i* in the crop (mg kg^−1^) and Q is the daily consumption of the crop in the experimental area (kg day^−1^) [35]. The per capita consumption of wheat grain by Chinese inhabitants is 0.2 kg day^−1^ [49].

#### Health risks related to micronutrients and heavy metals in wheat grain

The dietary intake estimates and health risks related to the levels of micronutrients and heavy metals through the consumption of wheat grains (Table 11) showed that, excluding Mn under all of the sheep manure application levels and Fe under the M4 treatment, the daily intake levels of the other micronutrients were lower than the recommended daily intake levels suggested by the Health and Human Services of the USA [48]. Thus, any deficiencies in Fe, Zn, and Cu among the local population should be compensated for by consuming foods rich in these nutrients. However, Mn may pose a potential threat to the health of the local population. The accumulated levels of heavy metals in wheat grain were lower than the recommended maximum tolerable levels, thereby suggesting that the risk to human health was still low.

## Conclusions

In general, the results of this long-term field experiment demonstrate that the application of sheep manure had no significant effects on the biomass and HI for winter wheat, or on the grain yield. The soil total N increased at a high compost application rate, but the available P was not affected. The available K was increased with most rates, where the average available K concentrations were 19% and 29% higher than the control during 2012 and 2013, respectively. The wheat straw and grain N concentrations tended to decrease with the compost treatments, where the average concentrations in 2012 and 2013 were reduced by 9% and 18% in straw, and by 16% and 12% in grain, respectively. The straw P concentration only increased in 2012, but the grain P increased in most cases. The straw K decreased in both years and the grain K only increased in 2013. The application of compost decreased the available Cu in the soil, but increased the concentrations of Fe, Zn, and Mn. In general, compost application enhanced the concentrations of Fe and Zn in wheat straw and grain, whereas the concentrations of Cu and Mn tended to decrease compared with the controls. The concentrations of Cd, Cr, and Pb were all at low levels, although their concentrations increased with some compost treatments. Cr was detected at 0.07–0.68 mg kg^–1^ in the edible parts of wheat, which was still much lower than the limit, but the levels increased greatly in winter wheat straw and grain when treated with sheep manure compost, which requires further investigation. The balances of macro- and micro-nutrients indicated that the removal of nutrients by wheat could be compensated for by the addition of compost, whereas the level of N decreased without the application of compost. The daily micronutrient intake rates due to the consumption of wheat grain obtained under the application of sheep manure compost were still lower than the recommended daily intake levels, except for that of Mn. The accumulated levels of heavy metals in wheat grain were also lower than the recommended maximum tolerable levels, thereby indicating that the levels of the heavy metals in the compost did not pose a potential threat to the safety of the soil and crop products.
